# Modelling of Nanoparticle Distribution in a Spherical Tumour during and Following Local Injection

**DOI:** 10.3390/pharmaceutics14081615

**Published:** 2022-08-02

**Authors:** George Caddy, Justin Stebbing, Gareth Wakefield, Xiao Yun Xu

**Affiliations:** 1 Department of Chemical Engineering, South Kensington Campus, Imperial College London, London SW7 2AZ, UK; g.caddy19@imperial.ac.uk; 2 Department of Surgery and Cancer, Imperial College London, London SW7 2AZ, UK; j.stebbing@imperial.ac.uk; 3 Xerion Healthcare Ltd., Cherwell Innovation Centre, 77 Heyford Park, Oxfordshire OX25 5HD, UK; gareth.wakefield@xerionhealthcare.co.uk

**Keywords:** mathematical modelling, radiotherapy, particle transport, tumour

## Abstract

Radio-sensitizing nanoparticles are a potential method to increase the damage caused to cancerous cells during the course of radiotherapy. The distribution of these particles in a given targeted tumour is a relevant factor in determining the efficacy of nanoparticle-enhanced treatment. In this study, a three-part mathematical model is shown to predict the distribution of nanoparticles after direct injection into a tumour. In contrast with previous studies, here, a higher value of diffusivity for charged particles was used and the concentration profile of deposited particles was studied. Simulation results for particle concentrations both in the interstitial fluid and deposited onto cells are compared for different values of particle surface charges during and after injection. Our results show that particles with a negative surface charge can spread farther from the injection location as compared to uncharged particles with charged particles occupying 100% of the tumour volume compared to 8.8% for uncharged particles. This has implications for the future development of radiosensitizers and any associated trials.

## 1. Introduction

Cancer is one of the leading causes of death worldwide, with over half of cases treated using radiotherapy. Radiotherapy involves the use of high-energy particles or waves to kill tumour cells [1]. There are two primary radiotherapy mechanisms: DNA targeting to cause strand breaks and thus no further cell replication; and the generation of highly reactive particles. These free radicals are created through the Compton scattering of X-rays off water molecules. Here, the scattering transfers energy to an electron that then scatters off other nearby electrons in a cascading effect before an interaction with an oxygen atom occurs. This results in a superoxide free radical that causes structural damage to nearby cell membranes resulting in apoptosis [1,2]. Damage is caused to all cells encountered by the radiation and so treatment is a balance between destroying the cancerous cells and minimising the damage to healthy cells [3].

As improving the efficacy of radiotherapy is an area of keen interest, radio-sensitizing nanoparticles are inert agents that can be directly injected into the tumour to increase the damage caused to the cancerous cells [2,4,5,6,7,8,9]. The nanoparticles increase the number of free radical particles by amplifying the generation of scattered electrons during the cascading effect. However, the exact distribution of nanoparticles within the tumour post-injection remains unclear, only that it will be heterogeneous [10]. The effect of radio-sensitizing particles is typically localized; therefore, the distribution of particles within the targeted tumour has a direct influence on treatment outcomes. It would be most desirable to achieve a uniform distribution of particles covering the entire tumour without spill-over into surrounding healthy tissue. To this end, the mathematical modelling of nanoparticle transport and fluid flow within a tumour can provide valuable insights that are difficult to measure with experimental techniques.

Computational and mathematical models have been used in previous studies to understand the transport processes involved in the delivery of chemotherapy drugs. These include investigating the effects of the tumour shape [11] and capillary network on drug delivery [12], improving realism through the use of realistic tumour geometry [13] and studying the effect of adjuvant therapies to improve drug delivery [14] among others. The transport of nanoparticles differs in that there can be a substantial deposition of particles onto cells, and this can have a significant effect on the final particle distribution. The addition of a deposition term to the equation governing particle transport within tumours has been derived through a variety of means across a number of different studies. Some studies set the deposition rate to be constant throughout the domain [15,16] or used previously developed semi-analytical correlation equations to calculate the deposition rate [17], and others developed their own particle trajectory tracking models [18,19].

**Figure 1:**
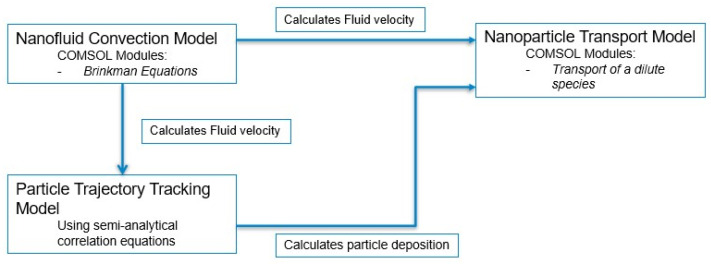
Schematic overview of the computational particle transport model.

In this work, a three-part computational particle transport model has been developed to predict the spatiotemporal concentration of nanoparticles during the direct injection of radio-sensitizing nanoparticles into a solid tumour. A schematic overview of the model is shown in Figure 1, where the first two parts calculate particle deposition and the interstitial fluid velocity, respectively; these are then inputted into the third part, a nanoparticle transport and deposition model. In this study, an idealised tumour geometry is used with a realistic needle inserted. This study investigates the distribution and concentration of particles within the fluid and is deposited onto cells throughout injection and following the end of injection. The effects of the particle surface charge and diffusion coefficient on the distribution of particles within the tumour are also investigated.

## 2. Materials and Methods

### 2.1. Mathematical Models

The mathematical model consists of three parts, which are described below.

#### 2.1.1. Particle Trajectory Tracking Model

The first part of the model calculates the rate of particle deposition onto cell surfaces, this can be calculated through [17]:

(1) $$k_{f}=\frac{3\left(1-\varepsilon \right)}{2\varepsilon d_{c}}\eta_{s}\left|u\right|$$

where ε is the porosity of the medium, $d_{c}$ is the diameter of the cells, |u| is the magnitude of the local fluid velocity, and $\eta_{s}$ is the collection efficiency that is defined as the ratio of particles that deposit on the cell surface to the total number of particles passing the cell. It is given by the expression [20]:

(2) $$\eta_{s}=\alpha \eta_{0}$$

where α is the attachment efficiency representing the effect of repulsive electrostatic forces on the fraction of particles colliding with the cells, while $\eta_{0}$ is the single collector contact efficiency that describes the fraction of particles colliding with cell surfaces due to diffusion, interception, and attractive inter-molecular forces. Both can be estimated by using semi-analytical correlations. The single collector contact efficiency can be expressed as [21]:

(3) $$\eta_{0}=\eta_{d}+\eta_{i}+\eta_{g}$$

where $\eta_{d}$ accounts for the transport due to diffusion and is given by:

(4) $$\eta_{d}=2.4A_{s}^{1/3}N_{R}^{-0.081}N_{Pe}^{-0.715}N_{vdW}^{0.052}$$

$\eta_{i}$ represents the transport due to interception:

(5) $$\eta_{i}=0.55A_{s}N_{R}^{1.675}N_{AT}^{0.125}$$

$\eta_{g}$ accounts for the transport due to gravitation:

(6) $$\eta_{g}=0.22N_{R}^{-0.24}N_{G}^{1.11}N_{vdW}^{0.053}$$

The non-dimensional coefficients are given, with descriptions, in Table 1. The attachment efficiency is given by [22]:

(7) $$\alpha =2.527\times 10^{-3}N_{LO}^{0.7031}N_{E1}^{-0.3121}N_{E2}^{3.5111}N_{DL}^{1.352}$$

with the non-dimensional coefficients also included in Table 1.

#### 2.1.2. Nanofluid Convection Model

The second part of the model is concerned with nanofluid convection within the tumour (treated as a porous medium), where Brinkman equations are used to solve for the fluid pressure and velocity [23]. The equations describe conservations of mass and momentum assuming incompressible, steady-state flow through a porous medium. The mass conservation equation states that the divergence of the fluid velocity, ∇u, is equal to the difference between the source, $\phi_{B}$, and sink, $\phi_{L}$, terms of fluid [24].

(8) $$\rho \nabla u=\phi_{B}-\phi_{L}$$

where ρ is the density of the fluid. The source term represents the fluid leakage from capillary vessels and the sink term represents fluid removed from the interstitium by the lymphatic system. As tumours generally have a non-functioning lymphatic system, this is assumed to be zero [25]. In this study, the capillary vessels are neglected. The momentum equation is given by:

(9) $$\frac{\rho }{\varepsilon }\left(\frac{\partial u}{\partial t}+\left(u\cdot \nabla \right)\frac{u}{\varepsilon }\right)=-\nabla p+\nabla \cdot \left[\frac{1}{\varepsilon }\left\{\mu \left(\nabla u+{\left(\nabla u\right)}^{T}\right)\;\frac{2}{3}\mu \left(u\cdot \nabla \right)I\right\}\right]-\left(\kappa^{-1}\mu \right)u+F$$

where μ is the dynamic viscosity of the fluid, κ is the permeability of the porous medium, p is the interstitial fluid pressure, and F accounts for any other forces.

#### 2.1.3. Nanoparticle Transport and Deposition Model

The third part, a nanoparticle transport model, calculates the spatiotemporal concentration of the nanoparticles within the tumour. The transport of nanoparticles in a porous medium is described by the convection–diffusion–reaction equation, which has generally been used to model the transport of macromolecular therapeutic particles [11,13,26]. For nanoparticle transport, the reaction term accounts for the deposition of nanoparticles onto the cell surface as this greatly affects particle concentration within the fluid [27]. The inclusion of a concentration-dependent deposition rate leads to the final convection–diffusion–deposition equation [18]:

(10) $$\frac{\partial C}{\partial t}=\nabla \cdot \left(D_{e}\nabla C\right)-\nabla \cdot \left(uC\right)-k_{f}\cdot C$$

where C is the molar concentration of the particles in the fluid, $D_{e}$ is the effective diffusivity of the nanoparticles, u is the fluid velocity calculated in the previous nanofluid convection model, and $k_{f}$ is the deposition rate coefficient of the particles. The expression $\nabla \cdot \left(D_{e}\nabla C\right)$ describes the particle diffusion, ∇·(uC) represents the particle convection, and $k_{f}\cdot C$ denotes the particle deposition onto the cells. The value for $D_{e}$ can be estimated using the equation for predicting particles diffusivity within a fluid [14], in this equation, the diffusivity is only dependent upon the particle diameter. A recently published study, however, preformed simulations of nanoparticle diffusion and demonstrated that the diffusivity of the nanoparticles was dependent upon both particle diameter and surface charge [28]. With the diffusivity increasing by four orders of magnitude when the surface charge was increased from 0 mV to −20 mV. This is due to the effect of electrostatic repulsion between the particles themselves. To account for the increase in diffusivity for charged particles, simulations of the negatively charged particles were run with the diffusivity being 10^−7^ m^2^/s.

### 2.2. Concentration of Deposited Particles

The concentration of particles depositing onto the cell surfaces of the porous medium is calculated by solving the following ODE throughout the model domain for all time steps:

(11) $$\frac{\partial C_{d}}{\partial t}=\frac{k_{f}\cdot C}{S_{v}}$$

Here, $C_{d}$ is the concentration of deposited particles, $k_{f}$ is the rate of particle deposition, C is the concentration of the particles within the fluid and $S_{v}$ is the specific surface area. The specific surface area is the total solid surface in a unit volume and is given by [29]:

(12) $$S_{v}=-\frac{6}{d_{c}}\varepsilon \ln\left(\varepsilon \right)$$

### 2.3. Model Setup

**Figure 2:**
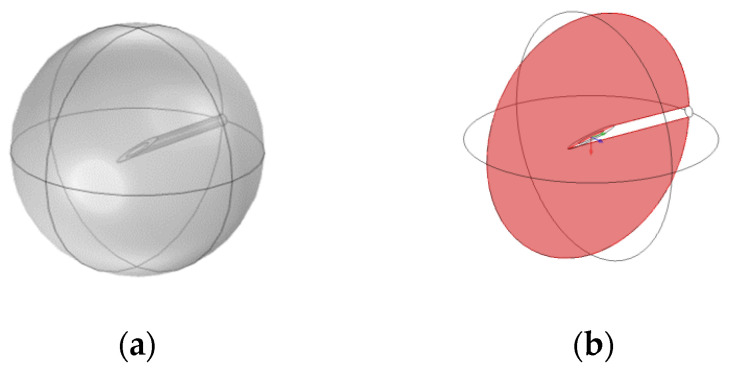
(**a**) Idealised tumour geometry with bevelled needle; (**b**) Cut plane through tumour, bisecting the needle along its shaft.

The tumour geometry is assumed to be an idealized sphere of radius 3.5 mm. The needle used to inject nanoparticles has realistic dimensions of a 26 s gauge bevelled tip needle [30], and the inlet of the needle is set to be at the centre of the tumour. Figure 2 shows the tumour geometry and a cut plane defined as bisecting the needle shaft. All sections of the model are implemented using COMSOL Multiphysics 5.6, COMSOL Inc., Stockholm, Sweden [31]. Meshing was done within COMSOL, with a finer mesh implemented around the needle tip.

The boundary conditions for different parts of the model are as follows: for the nanofluid convection model, a constant velocity is set at the inlet, the needle tip, and during injection based on the infusion rate and cross-sectional area of the needle, whereas at the outlet, the edge of the tumour, the pressure is set to zero. For the nanoparticle transport model, during injection, at the inlet the particle concentration is set to be constant, simulating continuous infusion. The outlet boundary condition for the particle concentration is a zero-flux condition at the outer edge of the tumour. When modelling after the end of injection the assumption is made that the velocity be zero throughout the domain and so the nanofluid convection model is not solved for this period. In reality, the velocity will become zero throughout the tumour within a short period of time following the end of injection, however, the significant reduction in computational cost offsets the slight simplification of this assumption. After the end of injection, the inlet in the nanoparticle transport model is converted into a no flux condition.

In addition, the nanofluid convection model is assumed to be steady, this is because the characteristic time for achieving steady state is approximately 0.05 s and the total time of injection is 10 s, this disparity allows for the assumption of steady-state interstitial fluid flow inside the tumour. Unless otherwise specified, all remaining simulation parameters used in the nanofluid convection and nanoparticle transport models are given in Table 2. The parameters used to calculate particle deposition from the correlation equations can be found in Table 3.

## 3. Results and Discussion

### 3.1. Particles with a Negative Surface Charge

**Figure 3:**
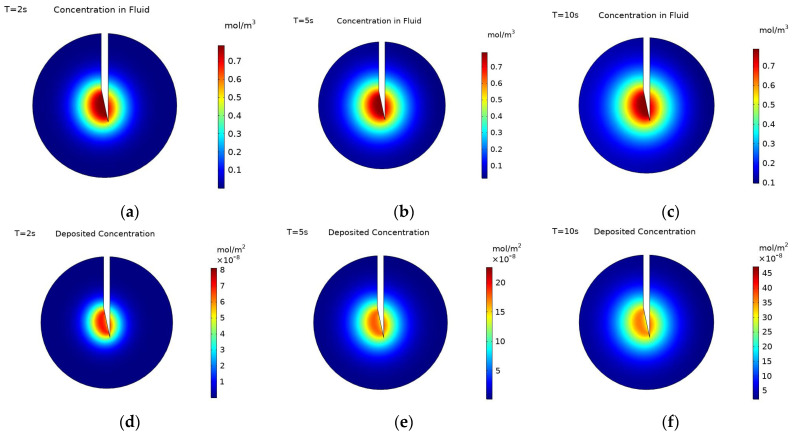
Concentration contours of charged particles (−20 mV) within the fluid at: (**a**) 2 s, (**b**) 5 s, and (**c**) 10 s during injection, and concentration contours of charged particles deposited onto cell surfaces at: (**d**) 2 s (**e**) 5 s, and (**f**) 10 s during injection. The cut plane is defined in Figure 2.

Figure 3 shows concentration contours at the cut plane (defined in Figure 1) for negatively charged nanoparticles both within the fluid and deposited onto cell surfaces during the injection at three time points: 2, 5, and 10 s, with 10 s being the end of injection. The maximum concentration of particles within the fluid does not change during the injection as this is constrained by the inlet concentration. Additionally, the spatial profile of the concentration does not change much during the injection, this is due to the transport mechanisms governing the movement of particles being in a quasi-equilibrium. Particles are moved away from the injection point by the convective force of the fluid velocity and additionally by the diffusivity of the particles. This is countered by the deposition of particles onto cell surfaces, acting to reduce the particle concentration throughout the tumour. There is some increase in particle concentration further from the injection point due to the high diffusivity of the particles, while convection will be insignificant far from the injection point.

**Figure 4:**
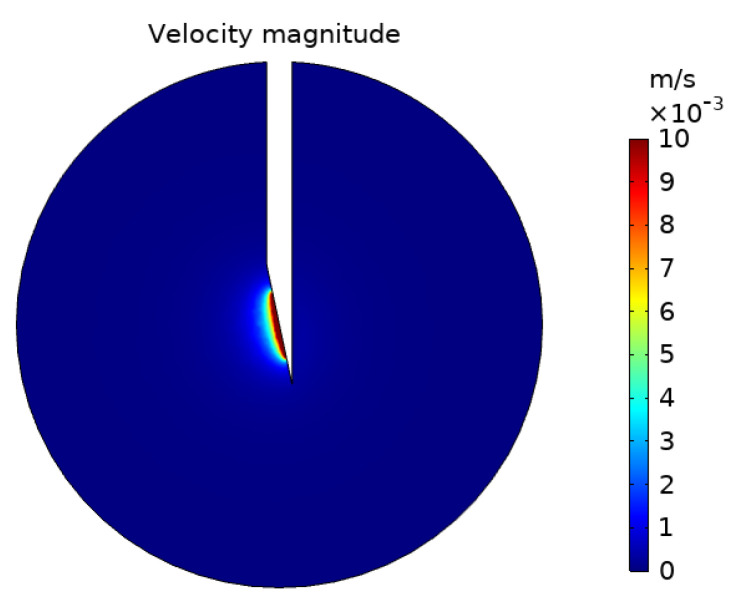
Fluid velocity magnitude contours at a cut plane through the tumour.

The concentration of deposited nanoparticles takes the units mol/m^2^ as it is the concentration of particles on the surface of the cells not the concentration of particles in a volume. The distribution of nanoparticles follows the same spatial pattern both within the fluid and on cell surfaces. The concentration of the particles deposited on cell surfaces is only dependent upon the concentration of particles within the fluid and the deposition rate at that point. Therefore, it is expected that the outer edge of the concentration profiles will follow the same pattern. There is variation, however, in the pattern of the concentration magnitude within the profiles. The concentration of particles within the fluid uniformly decreases when moving away from the injection point. This is due to the decrease in the convective velocity moving particles away from the needle tip and an increase in particle deposition, as deposition is inversely proportional to the fluid velocity. Whereas the concentration of deposited particles increases and then decreases when moving away from the injection location. This is because more nanoparticles pass the cells that are closer to the needle tip during injection, leading to a higher concentration of deposited particles there. However, as the rate of particle deposition is inversely proportional to the fluid velocity, fewer particles deposit immediately close to the needle tip where the fluid velocity is substantially higher. The magnitude of the fluid velocity decreases quickly when moving out from the needle, as shown in Figure 4. With the region of substantially high velocity corresponding entirely to the region of lower particle concentration in the immediate vicinity of the injection point. Beyond this, the velocity has fallen sufficiently that the rate of particle deposition ceases to be the dominant factor determining the concentration pattern. The distribution of deposited particles is not studied as frequently as the distribution of particles within the fluid [17,32,33], but as both will influence the efficacy of radiotherapy it is equally important to examine the distributions of both.

**Figure 5:**
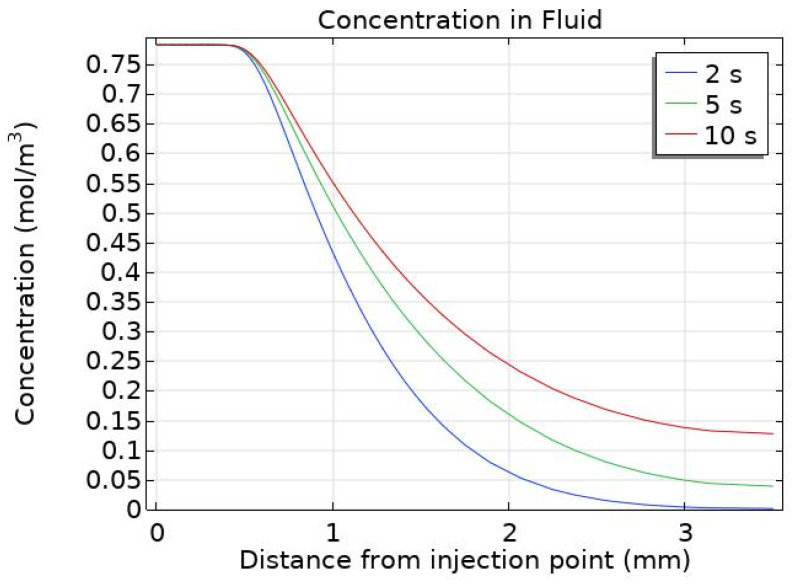
Particle concentration within the fluid during injection at 2 s, 5 s, and 10 s, along the distance from the point of injection.

The main difference between the three selected time points (at 2, 5, and 10 s) is the magnitude of the concentration; the spatial distribution of particles does not vary much beyond 5 s, but the magnitude of concentration still increases. This is clearly seen for the deposited particles with the maximum deposited concentration increasing from 8.30 × 10^−8^ mol/m^2^ at 2 s to 2.31 × 10^−7^ mol/m^2^ at 5 s to 4.83 × 10^−7^ mol/m^2^ at 10 s. The concentration of particles within the fluid is constrained by the inlet concentration and so the maximum concentration does not increase from 2 to 10 s. Figure 5 shows changes in the concentration of particles within the fluid along the distance from the point of injection for the three selected time points. It can be seen that although the concentration close to the injection point remains the same, the concentration further away is constantly increasing during the injection. This is due to the high diffusivity of the particles; away from the injection point the fluid velocity is minimal and so the transport of particles is due to diffusion alone. Although deposition is higher in regions where the velocity is low, the diffusion is strong enough to continue to transport particles away from the injection point, increasing the particle concentration there. This increase in concentration far from the injection point only occurs due to the increased value of diffusivity used for charged particles, something not considered in previous studies [10,17,19].

**Figure 6:**
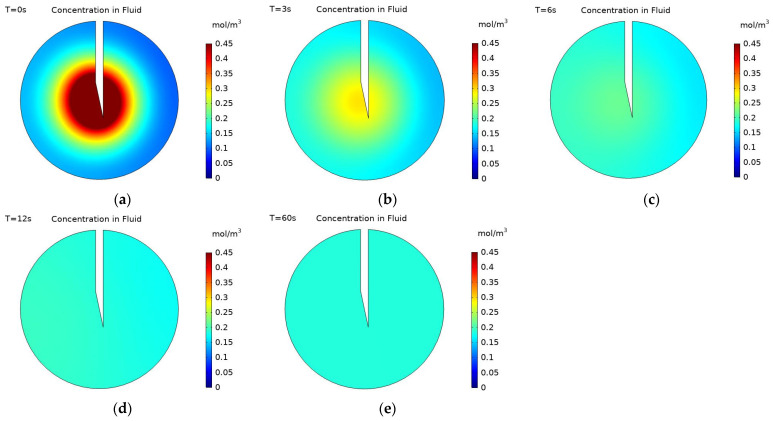
Concentration contours of charged nanoparticles (−20 mV) within the fluid at; (**a**) end of injection, and (**b**) 3 s, (**c**) 6 s, (**d**) 12 s, and (**e**) 60 s post-injection.

Figure 6 shows concentration contours for the distribution of nanoparticles within the fluid for five different time points: the end of injection, and 3, 6, 12, and 60 s post-injection. Here, the range of concentration values displayed in the figure has been restricted to allow for an easier comparison of the magnitude of concentration throughout the time period. It can be seen that after the end of injection, the concentration profile for particles in the fluid quickly becomes uniform across the tumour, with the concentration becoming 0.184 mol/m^3^ throughout the domain by 12 s post-injection. The concentration profile is unchanged from 12 s to 60 s post-injection as the concentration is uniform and diffusion no longer has any effect. There is no convective velocity after the end of injection and the deposition rate of particles is dependent upon the fluid velocity, as this is zero so too is the deposition rate. As the concentration of deposited particles is dependent on the deposition rate, the concentration of deposited particles remains constant from the end of injection until the end of the simulation.

**Figure 7:**
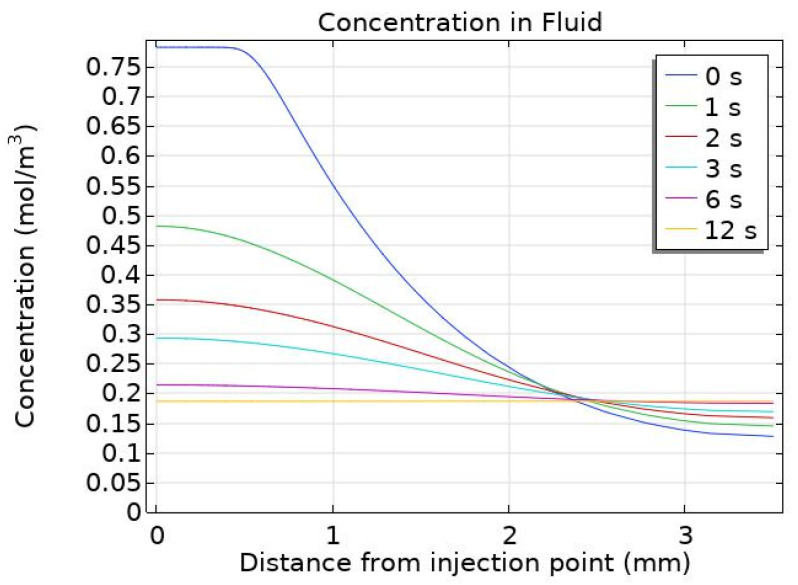
Particle concentration within the fluid during the first 10 s post-injection along the distance from the point of injection.

The nanoparticle concentration within the fluid remains constant from 12 s post-injection to the end of the simulation. However, within the first 12 s post-injection, the maximum particle concentration decreases dramatically from 0.784 mol/m^3^ to 0.184 mol/m^3^. Figure 7 displays changes in fluid particle concentration at six time points within the first 12 s post-injection; here, the effect of particle diffusivity on the maximum particle concentration is clear. The high effective diffusivity enables nanoparticles in areas of high concentration to move quickly to areas of low concentration, thereby achieving a uniform distribution across the tumour in less than 12 s post-injection.

**Figure 8:**
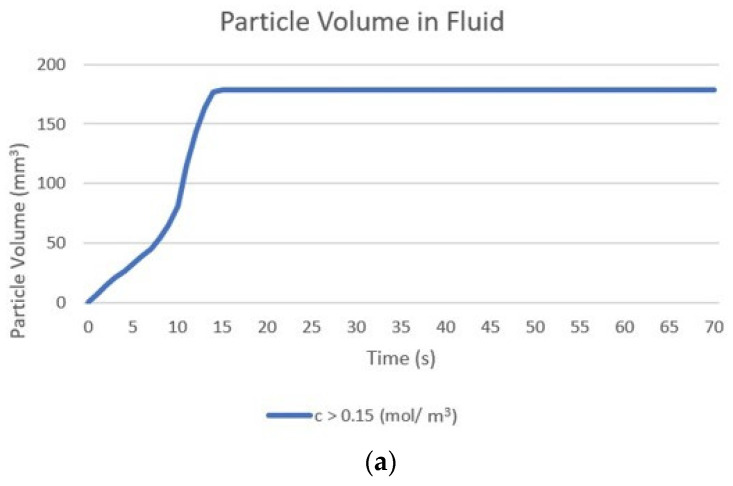

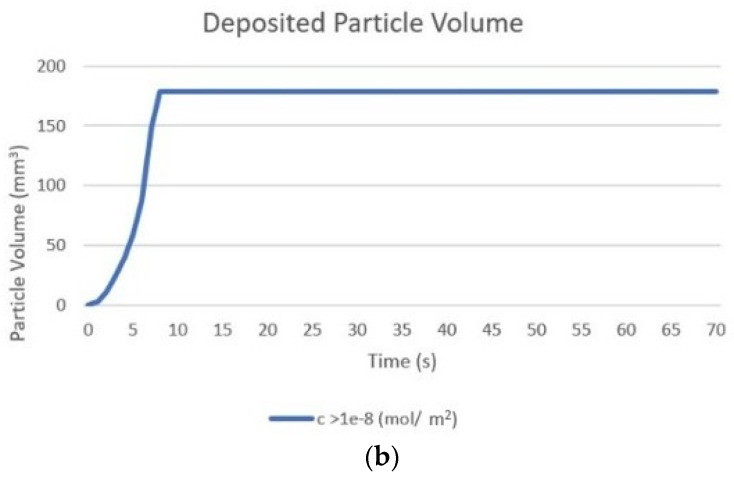
Change in the calculated volume of particles from the start of injection to 1 min post-injection particle. Particle volume is calculated as the sum of volumes where particle concentration is greater than the specified threshold: (**a**) volume within the fluid, threshold 0.15 mol/m^3^; (**b**) deposited onto cell surfaces, threshold 1 × 10^−8^ mol/m^2^.

For the quantitative evaluation of particle volumes within the tumour, a volume integration was performed using a threshold limit of concentration to set the edge of the volume containing particles. Figure 8 shows changes in the calculated volume of particles within the fluid and deposited onto cell surfaces from the start of the injection until 1 min post-injection, with the concentration thresholds being 0.15 mol/m^3^ for particles within the fluid and 1 × 10^−8^ mol/m^2^ for the deposited particles. It can be seen that the particle volume increases during the injection as the convective velocity and diffusion causes the particles to spread away from the needle tip. After the injection ends, the distribution of nanoparticles quickly becomes uniform across the tumour driven by their high diffusivity, and the redistribution of particles causes the concentration further from the injection point to increase above the threshold limit and so the particle volume becomes that of the entire tumour. The volume remains constant until the end of the simulation as there is no further particle transport once the concentration has become uniform. Figure 8b shows the change in the deposited particle volume, as previously discussed the volume of the deposited particles depends entirely on the volume of the particles within the fluid and so the deposited volume also increases rapidly during injection. Beyond this point, no change in particle volume is seen, as particle deposition is zero after the end of the injection.

### 3.2. Particles with Zero Surface Charge

**Figure 9:**
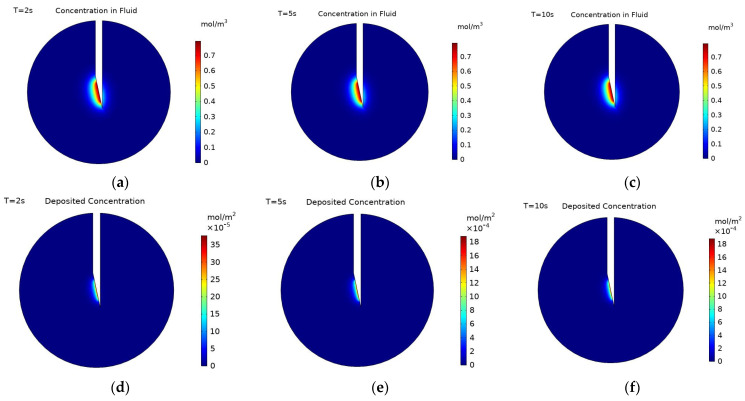
Concentration contours of uncharged nanoparticles within the fluid at; (**a**) 2 s, (**b**) 5 s, and (**c**) 10 s during injection, and concentration contours of uncharged particles deposited onto cell surfaces at; (**d**) 2 s (**e**) 5 s, and (**f**) 10 s during injection.

The particle surface charge has been shown to be a very important parameter in determining the final spread of nanoparticles within a tumour post-injection, both in previous computational and experimental studies [1,2,10]. Figure 9 shows concentration contours for the distribution of uncharged nanoparticles both within the fluid and deposited onto cell surfaces during the injection at 2, 5, and 10 s. In contrast to the negatively charged particles, the concentration profile of the particles within the fluid is completely restricted to the immediate vicinity of the needle tip, with the profile unchanging from 2 s onwards. This is because uncharged particles have a small diffusivity, and away from the injection site, once the movement of particles by the convective fluid velocity balances with the deposition of particles onto cell surfaces, the concentration profile will not change until after the end of the injection. The concentration of the deposited particles similarly does not change spatially during the injection, but the magnitude of the concentration does continue to increase, with the maximum concentration increasing from 4.15 × 10^−4^ mol/m^2^ at 2 s to 2.08 × 10^−3^ mol/m^2^ at 10 s. The substantial difference in particle distribution pattern between the negatively charged particles and the uncharged particles is due to the change in the deposition rate and particle diffusivity. With a substantial increase in the deposition rate for the uncharged particles compared to the negatively charged particles, the number of particles remaining in the fluid is significantly reduced, resulting in a decline in particle concentration away from the needle tip. In addition, the effective diffusivity of the uncharged particles is four orders of magnitude smaller than the negatively charged particles, significantly reducing the ability of the particles to move away from the needle tip and resulting in a spatially restricted concentration profile. As the profile of the deposited particles is dependent upon the profile of the particles within the fluid, this also sees a substantial reduction in the spread of the particles. However, the magnitude of the concentration is significantly higher than for the negatively charged particles because the uncharged particles are confined to a much smaller area.

**Figure 10:**
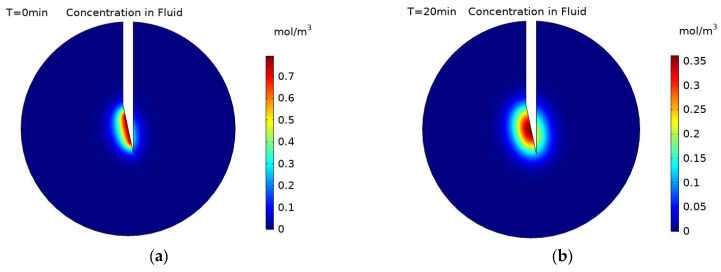

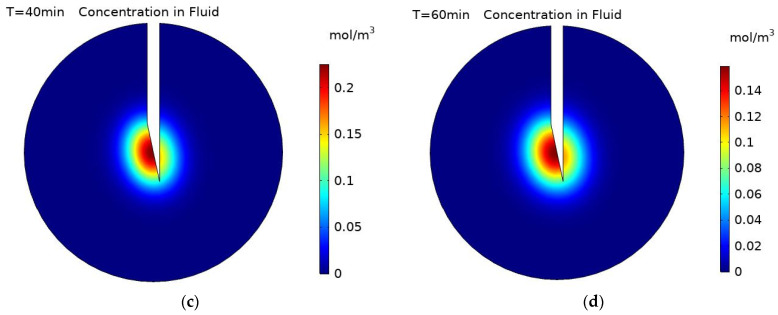
Concentration contours of uncharged nanoparticles within the fluid at; (**a**) 0 min, (**b**) 20 min, (**c**) 40 min, and (**d**) 60 min post-injection.

As the diffusivity of the uncharged particles is significantly lower, the time taken for the concentration of the particles in the fluid to become uniform post-injection is greatly increased as compared to the negatively charged particles. Figure 10 shows the distribution of the particles within the fluid at 20 min intervals from the end of the injection until 1 h post-injection. The high concentration area gradually expands over this period of time due to the particles’ diffusion but at a much slower rate than for the negatively charged particles as there is a four orders of magnitude difference between their diffusivities. As the particle deposition is zero after the end of injection, there is no change the concentration profile of deposited particles.

**Figure 11:**
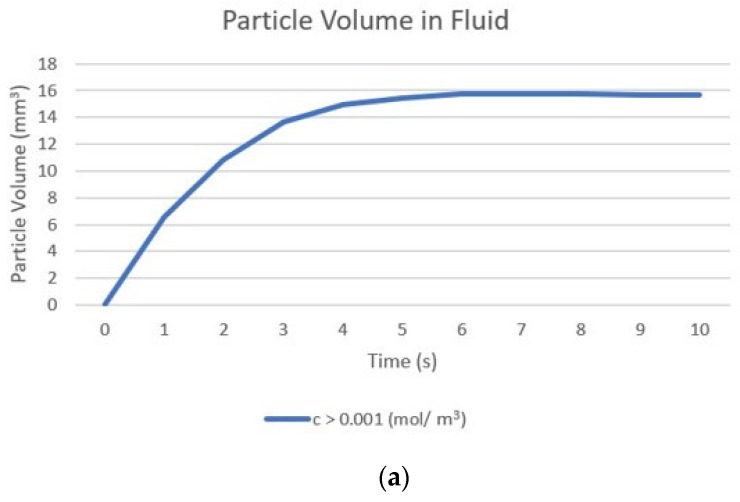

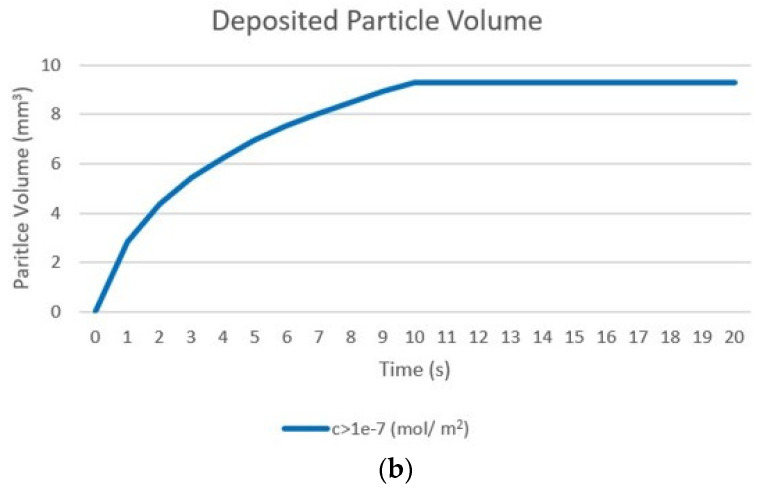
Change in the calculated volume of particles from the start of injection to 10 s post-injection: (**a**) within the fluid, threshold 1 × 10^−6^ mol/m^3^; (**b**) deposited onto cell surfaces, threshold 1 × 10^−9^ mol/m^2^.

Figure 11 shows the calculated volume of the nanoparticles within the fluid over time from the start of the injection to the end of the injection and the concentration of particles deposited onto cell surfaces until 10 s post-injection. This shows that the rate of increase in the particle volume within the fluid continually decreases until 7 s, then the volume remains constant until the end of the injection. The shape of the concentration profile is determined by the balance of the two main transport mechanisms: convection, and deposition. Close to the needle tip the convective velocity is sufficiently large to move particles away from the needle; as the fluid velocity declines the rate of deposition increases. The fraction of particles depositing onto a cell surface is inversely proportional to the fluid velocity, reaching a point where the volume of the particles no longer increases as these two competing mechanisms reach a state of equilibrium. The volume of the deposited particles increases during the injection, following the same pattern as that of the particles within the fluid, and then remains constant after injection as no further deposition occurs.

## 4. Conclusions and Future Perspectives

A three-part computational model to predict the spatio-temporal nanoparticle concentration has been developed. The model calculates the fluid velocity and nanoparticle deposition rate and uses these as inputs to a modified convection–diffusion equation giving the concentration of particles within the fluid and those deposited onto cell surfaces throughout the tumour over time. The concentration of the deposited particles has not been discussed in previous works [17,32,33], but is important for understanding the transport and distribution of nanoparticles. The spatial distribution of nanoparticles within the tumour is dependent upon the balance of the three transport mechanisms, fluid convection, particle diffusion, and particle deposition. For negatively charged particles during injection, convection and diffusion are more significant than deposition, with particles successfully moving away from the injection point. After injection, only diffusion still acts, causing the magnitude of the particle concentration in the fluid to become uniform across the tumour. Simulation results for uncharged particles have shown a significant reduction in the overall spread of particles away from the injection location, both for particles within the fluid, 178.7 mm^3^ to 15.7 mm^3^, and those deposited onto cell surfaces, 178.7 mm^3^ to 9.3 mm^3^, due to both their much lower diffusivity and increased rate of deposition compared to the negatively charged particles. The effect of particle surface charge on effective diffusivity has been investigated by Yuan et al. [27], who reported that the surface charge of nanoparticles can have a significant effect on the magnitude of their diffusivity; negatively charged nanoparticles were shown to have a diffusivity 10,000 times greater than uncharged particles. To the author’s knowledge, there have been no studies that take into account this variation in diffusivity when analyzing the effect of varying nanoparticle surface charge on the final nanoparticle distribution.

Several other factors can also influence the spatial distribution of nanoparticles in solid tumours, including the shape and size of the tumour [11], the tumour characteristics [14,34], the location of injection site [35,36], the number of injections [32,33], the nanoparticle concentration [37], and the injection rate [10,17], etc. The current model is limited to idealised geometry, a single injection point, and a simplified characterization of the tumour material. In the future, the model will be extended to account for a realistic shape of the tumour and its deformability. The influence of the injection location and the injection rate on the final particle concentration profile will also be investigated.

## Figures and Tables

**Table 1:** Definitions of non-dimensional coefficients used in correlation equations. All symbols are defined in Table 3.

Coefficient	Value	Description
$A_{s}$	$\frac{2\left(1-\gamma^{5}\right)}{\left(2-3\gamma +3\gamma^{5}-2\gamma^{6}\right)}\;\gamma ={\left(1-\varepsilon \right)}^{1/3}$	Porosity-dependent parameter of Happel model
$N_{R}$	$\frac{d_{p}}{d_{c}}$	Aspect ratio
$N_{Pe}$	$\frac{Ud_{c}}{D_{p}}$	Peclet number
$N_{vdW}$	$\frac{A_{H}}{k_{B}T}$	van der Waals number
$N_{AT}$	$\frac{A_{H}}{12\pi \mu r_{p}^{2}U}$	Attraction number
$N_{G}$	$\frac{2}{9}\frac{r_{p}^{2}\left(\rho_{p}-\rho_{f}\right)g}{\mu U}$	Gravity number
$N_{LO}$	$\frac{A_{H}}{9\pi \mu a_{p}^{2}U}$	London number
$N_{E1}$	$\frac{\varepsilon_{r}\varepsilon_{0}\left(\xi_{p}^{2}+\xi_{c}^{2}\right)}{6\pi \mu r_{p}U}$	First electrokinetic parameter
$N_{E2}$	$\frac{2\xi_{p}\xi_{c}}{\xi_{p}^{2}+\xi_{c}^{2}}$	Second electrokinetic parameter
$N_{DL}$	$2\kappa r_{p}$	Double Layer Force parameter

**Table 2:** Properties and parameters for the nanofluid convection and nanoparticle transport models (Data extracted from [18]).

Parameters and Properties	Value
Injection Amount	0.2 cc
Injection Rate	20 × 10^−4^ L/s
Needle	26 gauge
Nanoparticle Concentration	0.783 mol/m^3^
Tumour Porosity	0.4
Tumour Permeability	5 × 10^−13^ m^2^
Fluid Density	960 kg/m^3^
Fluid viscosity	1 × 10^−3^ kg/(ms)
Nanoparticle diffusivity	10^−11^ m^2^/s (0 mV) 10^−7^ m^2^/s (−20 mV)
Time step	0.1 s (during injection), 1 s (after injection)

**Table 3:** Properties and parameters for the correlation equations (Data extracted from [18]).

Parameters and Properties	Value
Tumour Porosity, ε	0.4
Fluid Density, $\rho_{f}$	960 kg/m^3^
Fluid Viscosity, μ	1 × 10^−3^ kg/(ms)
Nanoparticle Density, $\rho_{p}$	1060 kg/m^3^
Cell Diameter, $d_{c}$	15 × 10^−6^ m
Cell Surface Charge, $\xi_{c}$	−20 mV
Nanoparticle Surface Charge, $\xi_{p}$	0, −20 mV
Particle Diameter, $d_{p}$	40 × 10^−9^ m
Fluid Velocity, U	1 × 10^−4^–1 × 10^−1^ m/s
Nanoparticle Diffusivity, $D_{p}$	1 × 10^−11^ m^2^/s (0 mV) 10^−7^ m^2^/s (−20 mV)
Hamaker Constant, $A_{H}$	4 × 10^−20^ J
Debye–Huckel Parameter, κ	4.51 × 10^6^ (m/mol)^1/2^
Temperature, T	310.15 K
